# Non-HDL-to-HDL cholesterol ratio predicts incident T2DM and CHD in non-alcoholic fatty liver disease: evidence from a large clinical cohort and NHANES

**DOI:** 10.3389/fnut.2026.1750177

**Published:** 2026-05-19

**Authors:** Yanqi Kou, Yanxuan Zhong, Qing Zhang, Zhuoyan Lu, Jietao Liu, Yajuan Chen, Juntao Liang, Siyao Xu, Shiqi Zhang, Zhiying Deng, Bohuan Lin, Shicai Ye, Jiayuan Wu, Yuping Yang

**Affiliations:** 1 Department of Gastroenterology, Affiliated Hospital of Guangdong Medical University, Guangdong Medical University, Zhanjiang, Guangdong, China; 2 Clinical Research Service Center, Affiliated Hospital of Guangdong Medical University, Zhanjiang, Guangdong, China

**Keywords:** coronary heart disease, mediation analysis, NHHR, nonalcoholic fatty liver disease, risk prediction, type 2 diabetes

## Abstract

**Backgrounds:**

Non-alcoholic fatty liver disease (NAFLD) is a common metabolic disorder linked to increased risk of type 2 diabetes mellitus (T2DM) and coronary heart disease (CHD). The Non-HDL-to-HDL Cholesterol Ratio (NHHR) is an emerging lipid marker, but its predictive value for T2DM and CHD in NAFLD patients is unclear. This study evaluated the association between NHHR and the incidence of T2DM and CHD in a large NAFLD cohort.

**Methods:**

We conducted a retrospective cohort study involving 13,741 NAFLD patients from the Affiliated Hospital of Guangdong Medical University (2018–2025) and validated our findings in NHANES cohort (1999–2018) comprising 5,789 individuals. Participants were stratified into quartiles, and multivariate logistic regression with restricted cubic splines assessed associations with incident T2DM and CHD, adjusting for confounders. Subgroup, interaction, and mediation analyses elucidated potential effect modifiers and mediators.

**Results:**

Higher NHHR was independently associated with an increased risk of T2DM in both cohorts (per unit increase: OR 1.08, 95% CI 1.06–1.11). A nonlinear dose–response relationship was observed, with a marked rise in T2DM risk at higher NHHR levels, and participants in the highest NHHR quartile had 67% higher odds of T2DM compared with the lowest quartile. Mediation analysis indicated that fasting glucose accounted for over half of the NHHR–T2DM association. In contrast, the association between NHHR and CHD was weaker and less consistent across cohorts, suggesting heterogeneity in its cardiovascular predictive value among patients with NAFLD. Findings for T2DM were robust across major subgroups and externally validated in NHANES.

**Conclusion:**

NHHR is a simple, readily available lipid index that independently predicts incident T2DM in patients with NAFLD. Its nonlinear risk pattern and partial mediation through glucose metabolism underscore its clinical utility for early risk stratification and personalized prevention strategies.

## Introduction

Nonalcoholic fatty liver disease (NAFLD) currently affects approximately 25% of adults worldwide and has become recognized as the hepatic component of the metabolic syndrome. Its development is primarily driven by obesity, insulin resistance, dyslipidemia, and chronic low-grade inflammation (1). Over the past decade, evidence has expanded beyond liver-related complications, demonstrating that NAFLD is a multisystem disorder impacting multiple organs and regulatory mechanisms (2, 3). Besides its progression to steatohepatitis, fibrosis, and cirrhosis (4), NAFLD significantly increases the risk of developing type 2 diabetes mellitus (T2DM) and coronary heart disease (CHD). These conditions are among the key extrahepatic complications that contribute to increased morbidity and mortality in affected populations (5, 6).

Traditional lipid parameters—namely low-density lipoprotein cholesterol (LDL-C), high-density lipoprotein cholesterol (HDL-C), and triglycerides—offer an incomplete picture of the intricate atherogenic environment (7). Recognizing this limitation, clinical guidelines have increasingly advocated for broader lipoprotein targets. Notably, in 2021, the UK’s National Institute for Health and Care Excellence (NICE) recommended non-HDL-C, rather than LDL-C alone, as the primary lipid marker for cardiovascular risk reduction in patients with diabetes. This shift highlights non-HDL-C’s superior ability to reflect the total atherogenic burden.^1^ Building on this approach, the Non-HDL-to-HDL Cholesterol Ratio (NHHR), calculated as (total cholesterol (TC) – HDL-C) divided by HDL-C, integrates all apoB-containing lipoproteins relative to HDL-mediated reverse cholesterol transport. Several studies have demonstrated that NHHR has enhanced prognostic utility for predicting the onset of diabetes, cardiovascular events, and even renal and neuropsychiatric outcomes across diverse populations (8–11).

Although promising data exist, the predictive utility of NHHR for the development of T2DM and CHD has not yet been specifically evaluated in patients with diagnosed NAFLD. This is a particularly relevant population, as both dyslipidemia and cardiometabolic risk are substantially heightened in these individuals. Most existing studies have either focused on general populations or patients with diabetes, often without accounting for hepatic status, thereby leaving a critical gap in understanding NHHR’s role within a disease characterized by profound lipid abnormalities (12, 13).

To address this gap, we conducted a longitudinal analysis involving 13,741 real-world NAFLD patients to assess whether baseline NHHR predicts the future development of T2DM and CHD. We further validated our findings in a nationally representative cohort from NHANES, integrating clinical and population-based evidence to rigorously evaluate NHHR’s potential as a dual-risk biomarker in NAFLD.

## Methods

### Data source

Participants were retrospectively included based on their attendance at the Affiliated Hospital of Guangdong Medical University from 2018 to 2025. Initially, 15,741 patients with NAFLD were screened for eligibility. Exclusion criteria included the presence of T2DM or CHD, missing data on total cholesterol or HDL-C tests, or a missing rate of key covariates exceeding 20%. After applying these criteria, 13,741 eligible NAFLD patients were included in the final analysis (detailed in the patient selection flowchart in the outcome section). Participants were stratified into four groups (Q1, Q2, Q3, and Q4) based on NHHR quartiles, with Q1 serving as the reference group.

**Figure 1:**
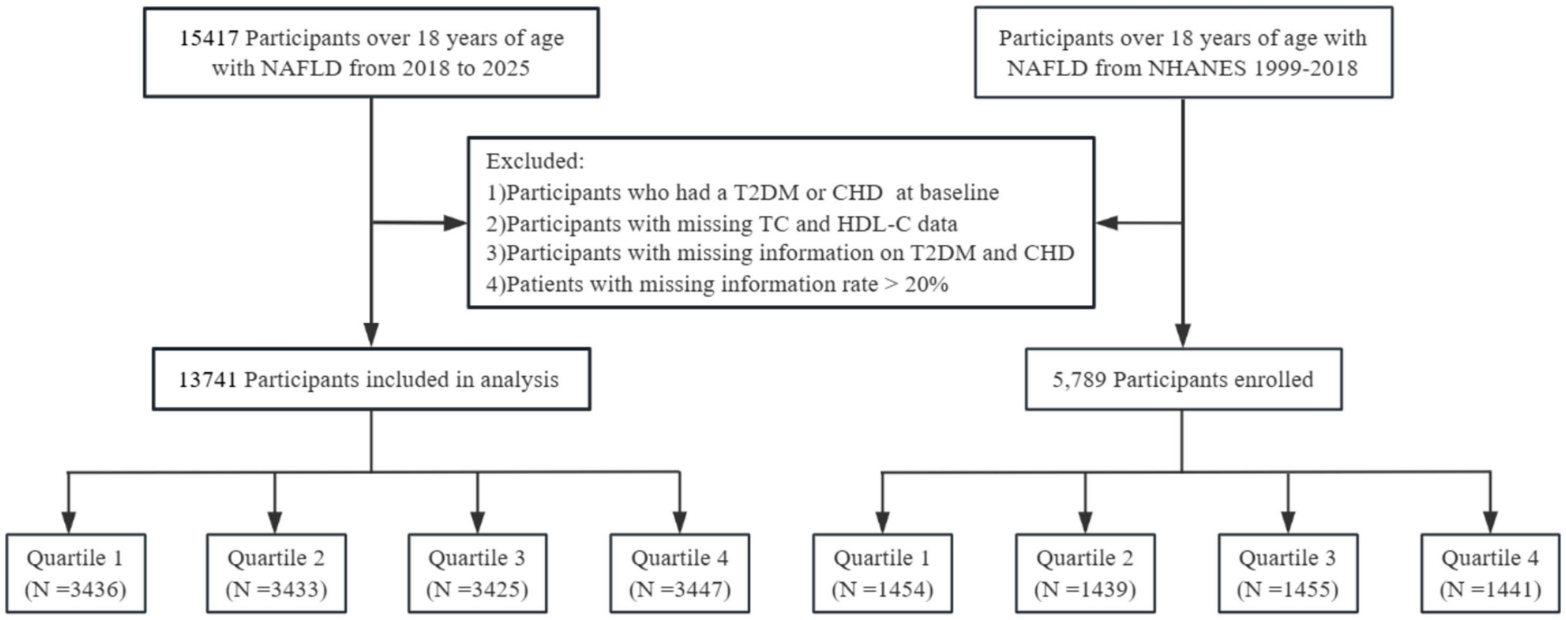
Study population selection flowcharts.

Data for external validation cohorts were obtained from NHANES. All protocols were reviewed and approved by the NCHS Research Ethics Review Committee, with informed consent obtained from each participant (or parent/guardian for minors).^2^ Data from 1999 to 2018 were used to identify NAFLD cases via the US-FLI (14, 15), excluding patients with other chronic liver diseases (e.g., viral hepatitis, excessive alcohol intake). A total of 5,789 eligible NHANES participants were included. Participants were stratified into quartiles (Q1–Q4) based on NHHR values (Figure 1).

### Variable definitions and measurements

The primary exposure factor, NHHR, was calculated as the ratio of non-high-density lipoprotein cholesterol to high-density lipoprotein cholesterol (non-HDL-C divided by HDL-C). Non-HDL-C was determined by subtracting HDL-C from TC. All lipid and biochemical indicators were measured from fasting blood samples collected either at hospital admission in the hospital cohort or during the NHANES mobile medical examination, using standard automated methods conducted in accredited clinical laboratories.

Covariate data were obtained from hospital records or NHANES interviews and examined variables such as age (continuous), gender (male/female), ABO blood type (A, B, AB, O), BMI (calculated as weight divided by height squared) both as a continuous and categorical variable (<24 kg/m^2^ vs. ≥24 kg/m^2^). Marital status was categorized as married/cohabiting or single (including unmarried, divorced, separated, widowed). Hepatitis B and hypertension diagnoses were based on medical records. Behavioral factors like smoking and alcohol use differed between cohorts: in the hospital data, these were from admission reports; in NHANES, smoking was defined by lifetime cigarette count and alcohol intake from dietary interviews, classified as drinkers or non-drinkers. Outcome measures included new T2DM and CHD, identified via clinical diagnosis in the hospital cohort and through HbA1c levels or self-reported diagnoses in NHANES.

### Statistical analyses

Continuous variables were reported as the means and standard deviations (SDs), while categorical variables were reported as frequencies and percentages. Group comparisons for continuous variables were performed using the Wilcoxon rank-sum test or Kruskal-Wallis test. For comparison between groups of categorical data, we used the Fisher exact test for expected frequencies <5; otherwise, we used the Chi-squared test.

We employed multiple linear regression and logistic regression analyses to examine the relationships between NHHR and T2DM and CHD. Three models were constructed to control for confounding factors: Model 1 without covariate adjustments; Model 2 adjusted for age, sex, and ABO blood type; and Model 3 further adjusted for BMI, marital status, smoking status, alcohol consumption, hepatitis B status, and hypertension. Additionally, restricted cubic spline (RCS) curve models analyzed the potential nonlinear dose–response relationship using NHHR as a continuous variable. The threshold effect was identified with piece-wise logistic regression and likelihood-ratio test. Subgroup and interaction analyses evaluated whether associations varied across factors such as BMI, gender, ABO blood group, marital status, smoking, alcohol intake, hepatitis B, hypertension, and disease history. Mediation analysis tested whether body mass index, fasting blood glucose, and inflammatory markers [white blood cell count (WBC) and neutrophil-to-lymphocyte ratio (NLR)] mediated the link between NHHR and outcomes, estimating direct, indirect, and total effects as well as the mediation proportion. To assess NHHR’s predictive value, receiver operating characteristic (ROC) curves were generated, and the area under the curve (AUC) quantified the added predictive power of NHHR. All analyses were conducted using R software (version 4.2.2), with *p* < 0.05 considered statistically significant.

## Results

### Baseline characteristics stratified by NHHR quartiles

The baseline characteristics of the study population stratified by NHHR quartiles are presented in Table 1, with participants divided into four groups based on their NHHR values: Q1 (<2.7), Q2 (2.7–3.55), Q3 (3.55–4.52), and Q4 (≥4.52). Significant differences were observed across these groups for most variables (*p* < 0.001), and participants in higher NHHR quartiles exhibited progressively worse metabolic and biochemical profiles. Specifically, BMI increased significantly from 25.1 ± 3.6 kg/m^2^ in Q1 to 26.0 ± 3.8 kg/m^2^ in Q4 (*p* < 0.001). The gender distribution showed a notable shift, with females constituting 65.8% of Q1 but only 36.8% of Q4, while males increased from 34.2 to 63.2% (*p* < 0.001). Lipid parameters demonstrated prominent quartile-dependent changes; HDL-C decreased by 39.3% from 1.40 ± 0.34 mmol/L in Q1 to 0.85 ± 0.22 mmol/L in Q4 (*p* < 0.001), whereas LDL-C increased by 47.8%, from 2.49 ± 0.78 mmol/L to 3.68 ± 1.46 mmol/L across the same quartiles (*p* < 0.001). Triglyceride levels nearly tripled, rising from 1.21 ± 0.66 mmol/L in Q1 to 3.20 ± 3.43 mmol/L in Q4 (*p* < 0.001), and fasting glucose levels increased from 5.43 ± 1.91 mmol/L to 6.00 ± 2.80 mmol/L (*p* < 0.001). Indicators of hepatic injury deteriorated progressively, with ALT levels rising by 37%, from 35 ± 77 U/L in Q1 to 48 ± 109 U/L in Q4 (*p* < 0.001), and GGT showing a 65% elevation from 52 ± 116 U/L to 86 ± 183 U/L (*p* < 0.001). The WBC count was highest in Q4 at 8.37 ± 7.81 × 10^9^/L compared to 7.82 ± 4.73 × 10^9^/L in Q1 (*p* < 0.001), indicating increased systemic inflammation.

**Table 1:** The baseline characteristics according to NHHR quartiles in the hospital cohort.

Characteristic	Quartiles of NHHR					*p*-value
	Overall *N* = 13,741	Q1 < 2.7 *N* = 3,436	Q2 (2.7–3.55) *N* = 3,433	Q3 (3.55–4.52) *N* = 3,425	Q4 ≥ 4.52 *N* = 3,447	
Age (year)	52 ± 14	51 ± 15	52 ± 14	53 ± 14	51 ± 14	<0.001
BMI (kg/m^2^)	25.6 ± 3.7	25.1 ± 3.6	25.5 ± 3.5	25.8 ± 3.6	26.0 ± 3.8	<0.001
Sex						<0.001
Female	7,189 (52.3%)	2,262 (65.8%)	1,960 (57.1%)	1,699 (49.6%)	1,268 (36.8%)	
Male	6,552 (47.7%)	1,174 (34.2%)	1,473 (42.9%)	1,726 (50.4%)	2,179 (63.2%)	
Marital status						0.160
Married	12,256 (89.2%)	3,068 (89.3%)	3,085 (89.9%)	3,062 (89.4%)	3,041 (88.2%)	
Single	1,485 (10.8%)	368 (10.7%)	348 (10.1%)	363 (10.6%)	406 (11.8%)	
Smoking						<0.001
No	12,452 (90.6%)	3,216 (93.6%)	3,185 (92.8%)	3,112 (90.9%)	2,939 (85.3%)	
Yes	1,289 (9.4%)	220 (6.4%)	248 (7.2%)	313 (9.1%)	508 (14.7%)	
Drinking						<0.001
No	12,992 (94.5%)	3,285 (95.6%)	3,272 (95.3%)	3,254 (95.0%)	3,181 (92.3%)	
Yes	749 (5.5%)	151 (4.4%)	161 (4.7%)	171 (5.0%)	266 (7.7%)	
ABO blood type						0.141
A	3,448 (25.1%)	809 (23.5%)	834 (24.3%)	877 (25.6%)	928 (26.9%)	
AB	912 (6.6%)	223 (6.5%)	232 (6.8%)	226 (6.6%)	231 (6.7%)	
B	3,701 (26.9%)	963 (28.0%)	928 (27.0%)	907 (26.5%)	903 (26.2%)	
O	5,680 (41.3%)	1,441 (41.9%)	1,439 (41.9%)	1,415 (41.3%)	1,385 (40.2%)	
Hypertension						0.089
No	11,019 (80.2%)	2,789 (81.2%)	2,772 (80.7%)	2,702 (78.9%)	2,756 (80.0%)	
Yes	2,722 (19.8%)	647 (18.8%)	661 (19.3%)	723 (21.1%)	691 (20.0%)	
Hepatitis B						<0.001
No	7,635 (55.6%)	1,793 (52.2%)	1,902 (55.4%)	1,922 (56.1%)	2,018 (58.5%)	
Yes	6,106 (44.4%)	1,643 (47.8%)	1,531 (44.6%)	1,503 (43.9%)	1,429 (41.5%)	
WBC (10^9^/L)	7.89 ± 5.33	7.82 ± 4.73	7.65 ± 4.33	7.70 ± 3.31	8.37 ± 7.81	<0.001
RBC (10^12^/L)	4.67 ± 0.70	4.53 ± 0.71	4.65 ± 0.66	4.75 ± 0.69	4.76 ± 0.72	<0.001
Hb (g/L)	130 ± 29	125 ± 29	130 ± 27	132 ± 28	132 ± 31	<0.001
PLT (10^9^/L)	262 ± 83	261 ± 87	259 ± 78	266 ± 84	264 ± 83	0.001
ALT (U/L)	38 ± 80	35 ± 77	33 ± 68	34 ± 56	48 ± 109	<0.001
AST (U/L)	30 ± 57	29 ± 55	27 ± 51	27 ± 41	35 ± 76	<0.001
GGT (U/L)	60 ± 127	52 ± 116	48 ± 87	53 ± 95	86 ± 183	<0.001
TC (mmol/L)	5.06 ± 1.32	4.28 ± 1.02	4.88 ± 0.97	5.23 ± 0.99	5.83 ± 1.67	<0.001
TG (mmol/L)	1.97 ± 2.00	1.21 ± 0.66	1.53 ± 0.74	1.92 ± 0.94	3.20 ± 3.43	<0.001
HDL-C (mmol/L)	1.12 ± 0.33	1.40 ± 0.34	1.18 ± 0.24	1.05 ± 0.20	0.85 ± 0.22	<0.001
LDL-C (mmol/L)	3.15 ± 1.12	2.49 ± 0.78	3.06 ± 0.83	3.37 ± 0.91	3.68 ± 1.46	<0.001
Glu (mmol/L)	5.63 ± 2.27	5.43 ± 1.91	5.48 ± 1.95	5.60 ± 2.24	6.00 ± 2.80	<0.001
CHD						0.046
No	13,268 (96.6%)	3,338 (97.1%)	3,310 (96.4%)	3,286 (95.9%)	3,334 (96.7%)	
Yes	473 (3.4%)	98 (2.9%)	123 (3.6%)	139 (4.1%)	113 (3.3%)	
T2DM						<0.001
No	11,978 (87.2%)	3,089 (89.9%)	3,056 (89.0%)	2,975 (86.9%)	2,858 (82.9%)	
Yes	1,763 (12.8%)	347 (10.1%)	377 (11.0%)	450 (13.1%)	589 (17.1%)	

All the variables are presented as the mean (SE) or *n* (%). NHHR, non-high-density lipoprotein cholesterol to high-density lipoprotein cholesterol ratio; BMI, body mass index; WBC, White Blood Cell Count; RBC, Red Blood Cell Count; Hb, Hemoglobin; PLT, Platelet Count; ALT, Alanine Aminotransferase; AST, Aspartate Aminotransferase; GGT, γ-Glutamyl Transferase; TC, Total Cholesterol; TG, Triglyceride; HDL-C, High-Density Lipoprotein Cholesterol; LDL-C, Low-Density Lipoprotein Cholesterol; Glu, Glucose; CHD, Coronary Heart Disease; T2DM, Type 2 Diabetes Mellitus.

Disease prevalence analysis revealed that T2DM increased significantly from 10.1% in Q1 to 17.1% in Q4, representing a 69% relative increase (*p* < 0.001), while CHD prevalence peaked in Q3 at 4.1% compared to 2.9% in Q1 (*p* = 0.046). There were no significant differences observed in marital status (*p* = 0.160), ABO blood type distribution (*p* = 0.141), or hypertension prevalence (*p* = 0.089). Similar patterns of adverse traits across NHHR quartiles were observed in an independent NHANES cohort (Supplementary Table S1), supporting the consistency of these associations across different populations.

### The correlation between NHHR and T2DM/CHD in NAFLD populations

NHHR as a continuous variable was significantly associated with an increased risk of T2DM across all models. Each unit increase in NHHR corresponded to an 8–9% higher risk (Model 1: OR 1.09, 95% CI 1.07–1.11, *p* < 0.001; Model 2: OR 1.09, 95% CI 1.07–1.11, *p* < 0.001; Model 3: OR 1.08, 95% CI 1.06–1.11, *p* < 0.001). When NHHR was categorized into quartiles, higher quartiles demonstrated progressively greater risks compared to the reference (Q1). In Model 1, Q3 (OR 1.35, 95% CI 1.16–1.56, *p* < 0.001) and Q4 (OR 1.84, 95% CI 1.59–2.12, *p* < 0.001) showed significant associations, which persisted after adjustment for covariates in Models 2 and 3 (Q4 in Model 3: OR 1.67, 95% CI 1.44–1.93, *p* < 0.001). A significant trend of increasing risk with higher NHHR quartiles was observed (*P* for trend < 0.001 in all models).

In the logistic regression analysis examining the association between NHHR and CHD risk, NHHR as a continuous variable showed no significant association across all models (Model 1: OR 1.00, 95% CI 0.97–1.03, *p* = 0.851; Model 2: OR 1.00, 95% CI 0.96–1.04, *p* = 0.827; Model 3: OR 0.99, 95% CI 0.95–1.03, *p* = 0.659). However, when NHHR was analyzed categorically, participants in the third quartile (Q3: NHHR 3.55–4.52) demonstrated a significantly higher risk of CHD compared to the reference group across all models (Model 1: OR 1.44, 95% CI 1.10–1.87, *p* = 0.007; Model 2: OR 1.35, 95% CI 1.03–1.75, *p* = 0.028; Model 3: OR 1.31, 95% CI 1.00–1.71, *p* = 0.046) (Table 2).

**Table 2:** The correlation between NHHR and T2DM/CHD in NAFLD populations in the hospital cohort.

Characteristic	Model 1			Model 2			Model 3		
	OR	95% CI	*P*-value	OR	95% CI	*P*-value	OR	95% CI	*P*-value
T2DM									
NHHR (continuous)	1.09	1.07, 1.11	<0.001	1.09	1.07, 1.11	<0.001	1.08	1.06, 1.11	<0.001
NHHR (quartile)									
Q1	1 (Reference)	1 (Reference)		1 (Reference)	1 (Reference)		1 (Reference)	1 (Reference)	
Q2	1.10	0.94, 1.28	0.237	1.07	0.92, 1.25	0.387	1.04	0.89, 1.21	0.630
Q3	1.35	1.16, 1.56	<0.001	1.30	1.12, 1.51	<0.001	1.23	1.06, 1.43	0.007
Q4	1.84	1.59, 2.12	<0.001	1.78	1.54, 2.06	<0.001	1.67	1.44, 1.93	<0.001
P for trend			<0.001			<0.001			<0.001
CHD									
NHHR (continuous)	1.00	0.97, 1.03	0.851	1.00	0.96, 1.04	0.827	0.99	0.95, 1.03	0.659
NHHR (quartile)									
Q1	1 (Reference)	1 (Reference)		1 (Reference)	1 (Reference)		1 (Reference)	1 (Reference)	
Q2	1.26	0.97, 1.66	0.088	1.21	0.92, 1.58	0.174	1.21	0.92, 1.59	0.172
Q3	1.44	1.10, 1.87	0.007	1.35	1.03, 1.75	0.028	1.31	1.00, 1.71	0.046
Q4	1.16	0.88, 1.52	0.298	1.09	0.82, 1.44	0.546	1.04	0.79, 1.38	0.773
P for trend			0.204			0.422			0.674

NHHR, non-high-density lipoprotein cholesterol to high-density lipoprotein cholesterol ratio; T2DM, Type 2 Diabetes Mellitus; CHD, Coronary Heart Disease; CI, Confidence Interval; OR, Odds Ratio.

Model 1: no covariates were adjusted.

Model 2: adjusted for sex, ABO Blood Type, and age.

Model 3: adjusted for sex, ABO Blood Type, age, BMI, marital status, smoking, drinking, hepatitis B, and hypertension.

In the NHANES cohort (Supplementary Table S2), the findings for T2DM were consistent with our main analysis: continuous NHHR remained a significant predictor of T2DM after full adjustment (Model 3: OR 1.09, 95% CI 1.04–1.14, *p* < 0.001), and participants in the highest quartile (Q4) had a 21% higher odds of T2DM compared to Q1 (OR 1.21, 95% CI 1.01–1.46, *p* = 0.043; *P* for trend = 0.049). However, the CHD results differed: in the NHANES data, a significant inverse association was observed between NHHR and CHD in Model 3 (OR 0.90, 95% CI 0.83–0.98, *p* = 0.021), indicating a protective effect. Specifically, the highest quartile (Q4) showed a lower risk for CHD compared to Q1 (OR 0.64, 95% CI 0.46–0.88, *p* = 0.006; p for trend = 0.006). The discriminative performance of the three models was further evaluated using ROC curve analysis, as shown in Supplementary Figure S1. NHANES showed a protective association between NHHR and CHD, which may be due to population differences or confounding factors.

### Detection of nonlinear relationships

**Figure 2:**
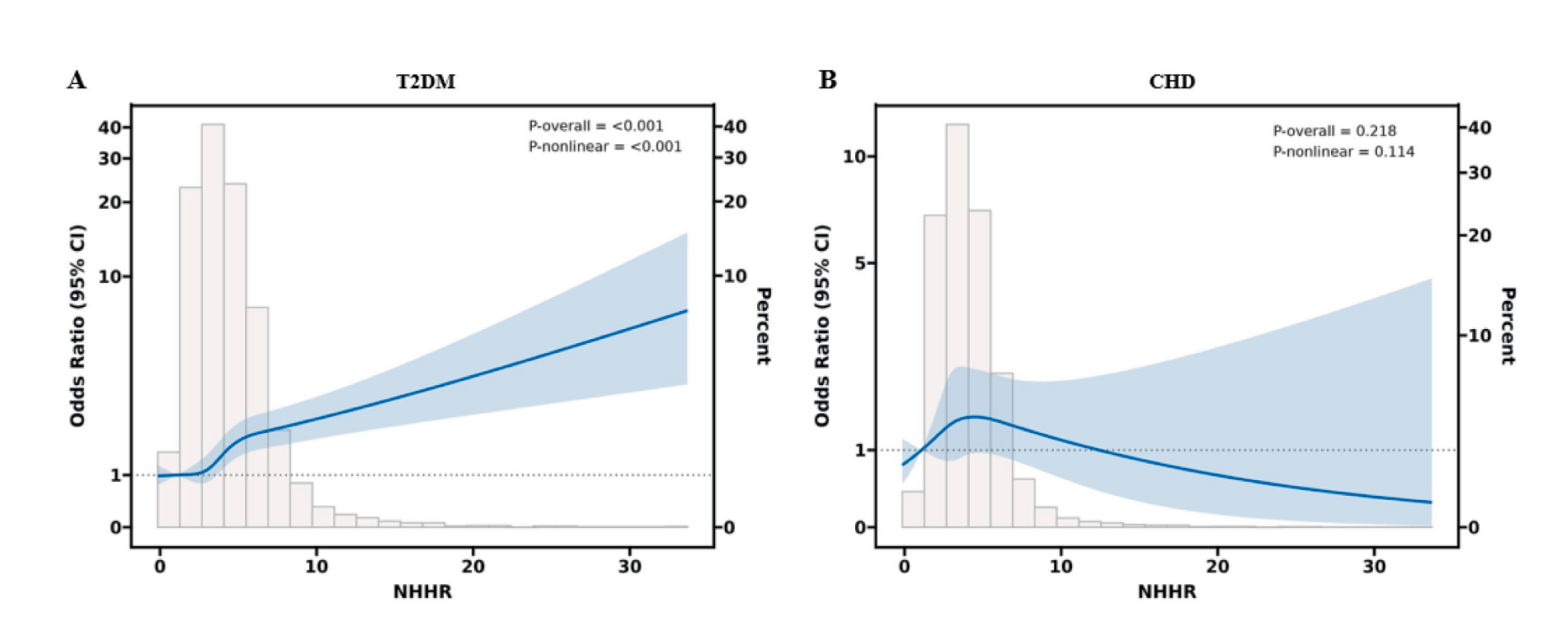
Restricted cubic spline analyses of NHHR associations with T2DM and CHD in the hospital cohort. **(A)** Nonlinear dose–response relationship between continuous NHHR and T2DM. **(B)** Nonlinear dose–response relationship between continuous NHHR and CHD.

The RCS curve model was used to further explore the possible nonlinear relationship between NHHR and T2DM and CHD (Figure 2). In our analysis, elevated NHHH was significantly associated with a nonlinear increase in diabetes mellitus risk. The RCS revealed a distinct nonlinear pattern, corroborating the threshold effect analysis that identified two inflection points at NHHH values of 3.89 and 5.66 (both *p* < 0.001). Below 3.89, the curve was nearly horizontal, suggesting minimal risk variation. A pronounced increase in the odds ratio was observed between 3.89 and 5.66, indicating a critical risk-sensitive range. Beyond 5.66, the slope of the curve decreased, with the odds ratio continuing to rise at a slower rate, implying partial saturation of risk at higher NHHH levels. The overall association was statistically significant (*p* < 0.001), and evidence of nonlinearity was strong (*p* < 0.001). Conversely, the relationship between NHHR and CHD was not statistically significant, and no discernible trend was observed.

### Subgroup analyses

**Figure 3:**
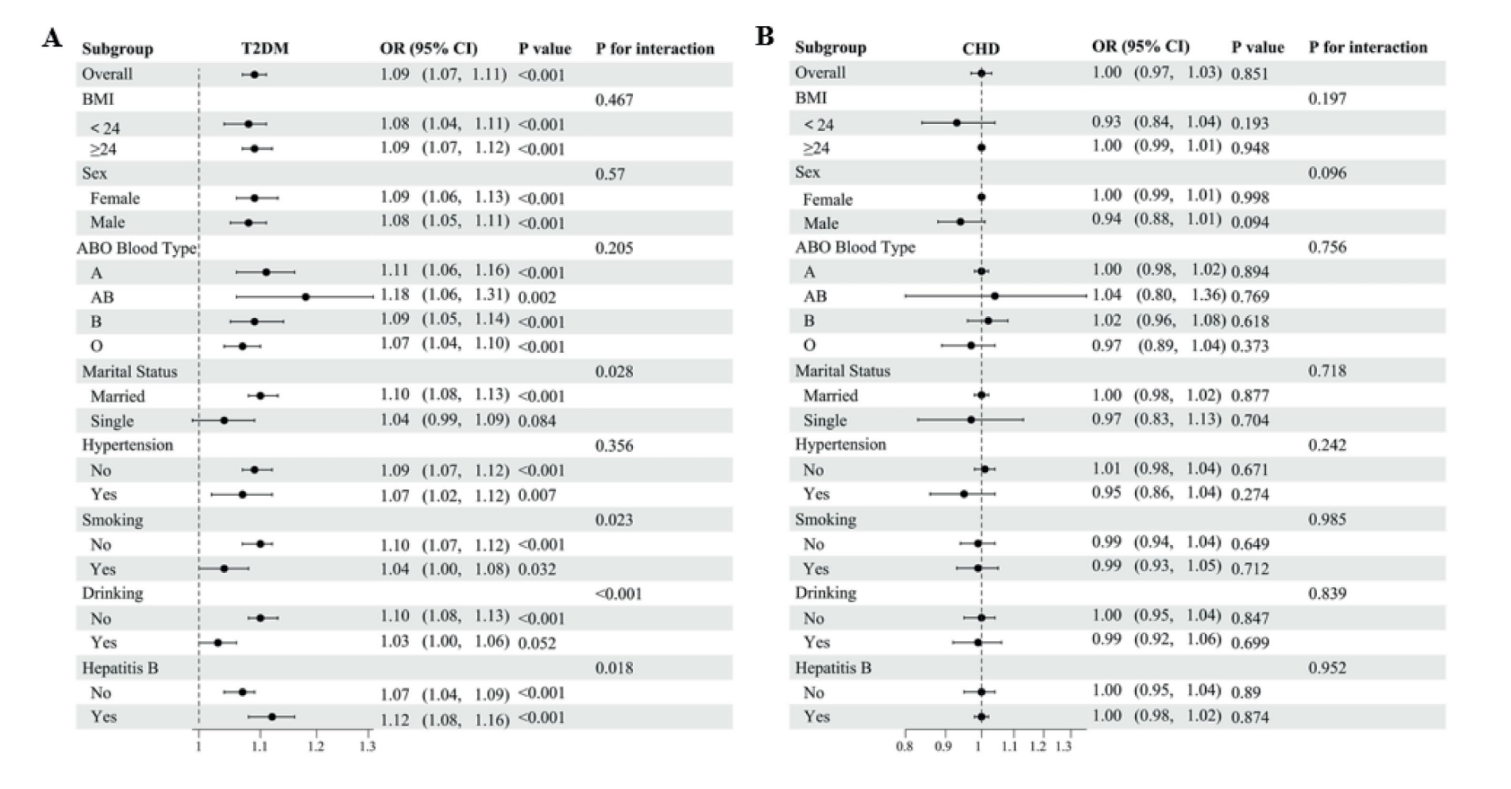
Forest plots of stratified analyses for the associations between NHHR and T2DM/CHD in the hospital cohort. **(A)** Subgroup analysis for T2DM. **(B)** Subgroup analysis for CHD.

To further explore the consistency of the association between NHHR and incident T2DM and CHD, we conducted subgroup and interaction analyses across BMI (<24 vs. ≥24 kg/m^2^), sex, ABO blood type (A, AB, B, O), marital status (married vs. single), hypertension, smoking, drinking and Hepatitis B infection status (Figure 3).

The overall analysis of 13,741 participants demonstrated a significant positive association between NHHR and DM (OR 1.09, 95% CI 1.07–1.11, *p* < 0.001). Subgroup analyses revealed consistent associations across BMI categories (BMI < 24: OR 1.08, 95% CI 1.04–1.11; BMI ≥ 24: OR 1.09, 95% CI 1.07–1.12, both *p* < 0.001) and sex (female: OR 1.09, 95% CI 1.06–1.13; male: OR 1.08, 95% CI 1.05–1.11, both *p* < 0.001), with no significant interaction effects (*P* for interaction = 0.467 for BMI, 0.57 for gender). Significant interactions were observed for marital status (*p* = 0.028), smoking status (*p* = 0.023), drinking status (*p* < 0.001), and HBsAg status (*p* = 0.018). Notably, stronger associations were observed among married individuals (OR 1.10, 95% CI 1.08–1.13) compared to single individuals (OR 1.04, 95% CI 0.99–1.09), and among non-smokers (OR 1.10, 95% CI 1.07–1.12) compared to smokers (OR 1.04, 95% CI 1.00–1.08). The association was particularly pronounced in HBsAg-positive individuals (OR 1.12, 95% CI 1.08–1.16) compared to HBsAg-negative individuals (OR 1.07, 95% CI 1.04–1.09).

In contrast, there was no significant association between NHHR and CHD (OR 1.00, 95% CI 0.97–1.03, *p* = 0.851), with no significant interactions detected across any stratification variables, and the associations remained consistently null across subgroups.

### Mediation analysis

**Figure 4:**
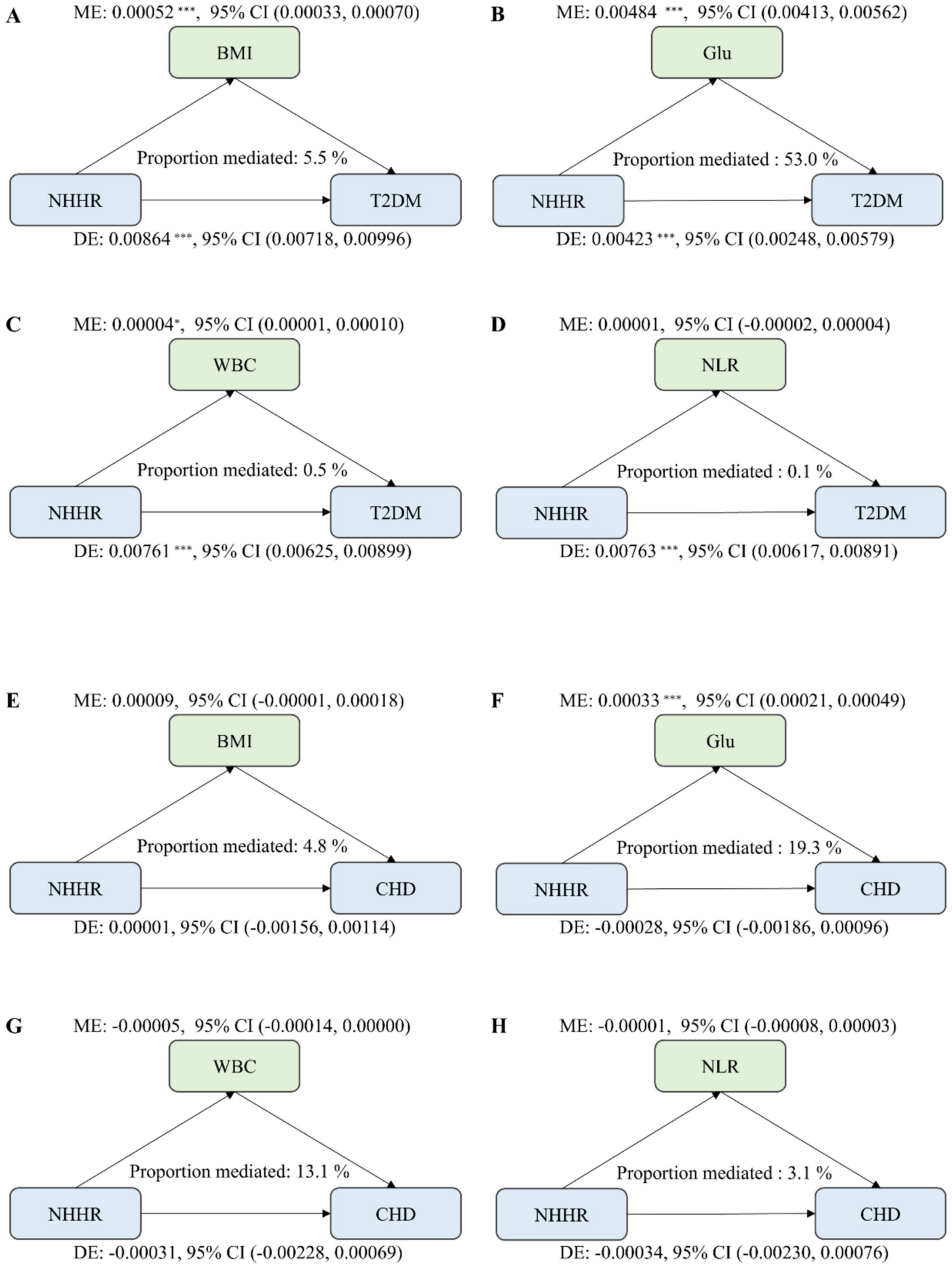
Mediation pathways in NHHR-disease relationships in the hospital cohort. **(A)** Mediation by BMI in the NHHR–T2DM pathway. **(B)** Mediation by fasting Glu in the NHHR–T2DM pathway. **(C)** Mediation by WBC in the NHHR–T2DM pathway. **(D)** Mediation by NLR in the NHHR–T2DM pathway. **(E)** Mediation by BMI in the NHHR–CHD pathway. **(F)** Mediation by fasting Glu in the NHHR–CHD pathway. **(G)** Mediation by WBC in the NHHR–CHD pathway. **(H)** Mediation by NLR in the NHHR–CHD pathway.

Finally, considering that BMI, fasting blood glucose, and inflammatory markers may serve as potential mediators in the relationship between NHHR and the development of T2DM and CHD, they likely reflect underlying metabolic disturbances, insulin resistance, and low-grade inflammation that could influence this association. Therefore, we employed mediation analysis to investigate the extent to which these factors mediate the relationship between NHHR and the risks of T2DM and CHD, aiming to elucidate their roles in the underlying pathophysiological mechanisms (Figure 4).

Regarding T2DM, the mediating effect of BMI demonstrated an indirect effect (IE) coefficient of 5.2 × 10^−4^ (95% CI, 0.00033–0.00070; *p* < 0.001), accounting for approximately 5.5% of the total effect. Glu showed an IE of 4.84 × 10^−3^ (95% CI, 0.00413–0.00562, *p* < 0.001), mediating approximately 53.0% of the total association between NHHR and T2DM. Similarly, WBC exhibited a small but statistically significant mediating effect (IE = 4 × 10^−5^; 95% CI: 0.00001–0.00010; *p* = 0.012), accounting for approximately 0.5% of the total effect, whereas NLR did not demonstrate a significant mediating effect (IE = 1 × 10^−5^; 95% CI: −0.00002 to 0.00004; *p* = 0.688). In contrast, the mediating role of BMI in the NHHR-CHD relationship was weak and not statistically significant (IE = 9.0 × 10^−5^; 95% CI: −0.00001 to 0.00018; indirect proportion = 4.8%). Conversely, Glu partially mediated the association between NHHR and CHD (IE = 3.3 × 10^−4^; 95% CI: 0.00021–0.00049; *p* < 0.001), accounting for approximately 19.3% of the total effect. For inflammatory markers, neither WBC (IE = −5 × 10^−5^; 95% CI: −0.00014 to 0.00000; *p* = 0.558) nor NLR (IE = −1 × 10^−5^; 95% CI: −0.00008 to 0.00003; *p* = 0.816) demonstrated a meaningful mediating effect in this pathway.

## Discussion

This study is the first to systematically evaluate the potential of NHHR as a predictor of T2DM and CHD risk among patients with NAFLD. Based on data from two independent cohorts—the real-world hospital cohort and the nationally representative NHANES database—our core findings demonstrate that higher NHHR levels are independently associated with a significantly increased risk of incident T2DM. This association exhibits clear dose–response and nonlinear characteristics. Notably, the relationship between NHHR and CHD was inconsistent across the two cohorts, suggesting that its predictive utility for CHD in the NAFLD population may be more complex. Mediation analysis further revealed that fasting blood glucose plays a critical mediating role in the NHHR-T2DM association, accounting for over 50% of the total effect. Overall, our results validate NHHR as a simple yet effective dual-risk biomarker with promising potential for predicting metabolic complications in NAFLD.

The discrepant associations between NHHR and CHD in the hospital cohort versus the NHANES population may reflect both methodological differences and underlying biological heterogeneity. These include differences in population characteristics, residual confounding, variations in outcome ascertainment, and limited statistical power due to relatively few CHD events, especially in subgroup analyses. More importantly, this inconsistency may reflect the context-dependent nature of NHHR as a risk marker. Cardiovascular risk is not determined by a single biomarker but arises from the interplay among lipid abnormalities, chronic inflammation, and vascular susceptibility (16). In clinically diagnosed NAFLD populations with a high baseline burden of metabolic dysfunction and inflammation, the additional risk signal captured by NHHR may be attenuated. In contrast, in general populations with lower baseline risk, NHHR may more sensitively reflect early lipid-driven risk perturbations. Therefore, the predictive performance of NHHR for CHD may depend on the underlying metabolic and inflammatory context, underscoring the need for context-specific interpretation in clinical practice. These findings further underscore the complexity of cardiovascular risk prediction in NAFLD and suggest that the clinical utility of NHHR for CHD risk assessment may be context-dependent rather than uniformly predictive.

NAFLD, T2DM, and CHD are prevalent chronic conditions that significantly impact public health worldwide. NAFLD affects approximately 25% of adults globally and is found in over half of people with T2DM, creating a powerful and dangerous interface between liver and cardiometabolic disease risk (1, 17). Meta-analyses have found that having NAFLD confers more than double the risk of incident T2DM (pooled hazard ratio ≈ 2.19, 95% CI 1.93–2.48) compared to those without NAFLD, and this risk rises in proportion to the severity of liver fibrosis (18). The relationship extends to cardiovascular disease: a global meta-analysis of 38 studies (*n* ≈ 67,000 NAFLD patients) reported a pooled CHD prevalence of 44.6% and a 33% higher odds of CHD (OR = 1.33, 95% CI 1.21–1.45) in NAFLD compared with non-NAFLD individuals, independent of traditional risk factors (19). Collectively, NAFLD, T2DM, and CHD are closely interrelated: NAFLD increases the risk of T2DM and CHD, while T2DM can exacerbate NAFLD progression. The coexistence of these conditions significantly raises cardiovascular morbidity and mortality (20, 21). The high prevalence and severe outcomes of these overlapping diseases highlight the urgent need for effective biomarkers like NHHR to identify high-risk NAFLD patients early and guide preventive measures.

Our results align with and extend prior work on NHHR’s prognostic value. In the NAGALA study of 15,464 Japanese adults, NHHR outperformed traditional lipid markers for incident diabetes—with an inflection around 2.74 (22). Additional NHANES analyses of 2007–2018 confirmed that NHHR was positively associated with T2DM risk, further supporting its role as a glycemic risk marker (12). Beyond diabetes incidence, higher NHHR was independently linked to major adverse cardiovascular events and all-cause mortality among patients with T2DM in multi-center cohorts (23). CHARLS and UK Biobank cohorts similarly associated higher NHHR with hypertension, ischemic heart disease, and cardiovascular outcomes in diabetic populations (24, 25). Importantly, NHHR has also shown strong performance in hypertension: cross-sectional NHANES analyses demonstrated its superiority to individual lipid parameters for predicting hypertension risk (26), while prospective NHANES follow-up revealed U-shaped associations of NHHR with all-cause and cardiovascular mortality in hypertensive adults, with thresholds around 2.32–2.65 (27). A PCI-patient cohort further identified a U-shaped NHHR–cardiac event curve centered near 3.12 (28). Notably, in metabolic dysfunction-associated steatotic liver disease, NHHR was associated with incident disease in a nonlinear fashion, with an inflection point at about 2.54, consistent across sensitivity analyses (29). Finally, in a recent analysis of 12,578 U. S. adults with diabetes or prediabetes using NHANES (1999–2018), restricted cubic spline models revealed a U-shaped association between NHHR and all-cause mortality and an L-shaped association for cardiovascular mortality, with thresholds at 2.72 and 2.83, respectively (30). Taken together, these data underscore NHHR as a broadly applicable, non-linear indicator of cardiometabolic risk, while our NAFLD sample uniquely suggests that CHD risk may plateau at very high ratios, possibly reflecting ceiling effects in a high-risk hepatic context.

NHHR likely reflects interrelated mechanisms linking dysregulated lipid metabolism, chronic low-grade inflammation, and insulin resistance in NAFLD to diabetes and atherosclerosis. Hepatic insulin resistance—driven by excess free fatty acids and *de novo* lipogenesis—increases VLDL–apoB secretion, elevating non-HDL-C and overwhelming HDL-mediated reverse cholesterol transport; this promotes lipotoxic intermediates that impair insulin signaling and β-cell function (31–33), and may also activate inflammatory signaling pathways that contribute to metabolic and vascular injury. The resulting atherogenic profile favors cholesterol delivery to the arterial intima, oxidative modification and foam-cell formation, effects amplified by NAFLD-related inflammation and oxidative stress (34). In our study, WBC showed a small but significant mediating effect in the NHHR–T2DM association, but not in CHD, while NLR was not significant in either outcome. This differs from previous evidence linking NLR to subclinical atherosclerosis in high-risk populations (35), suggesting that the role of inflammation may vary across markers, populations, and outcomes, and that NHHR may primarily reflect lipid-driven pathways in this context. Adipokine imbalance (low adiponectin, high leptin/resistin) exacerbates insulin resistance and perturbs lipoprotein metabolism (36, 37). Altered gut microbiota and increased permeability promote endotoxemia and TLR-mediated inflammation, further linking dyslipidemia to cardiometabolic risk (38, 39). By integrating non-HDL-C and HDL-C, NHHR reflects key pathophysiological processes such as lipotoxicity, insulin resistance, inflammation, and endothelial dysfunction, providing a mechanistic basis for its predictive utility. As a ratio of atherogenic to anti-atherogenic lipids, NHHR captures the balance between cholesterol delivery and reverse transport more effectively than individual measures. Empirically, it demonstrates stronger, more consistent associations with cardiometabolic outcomes, enhancing risk discrimination. Additionally, NHHR is easily derived from routine lipid panels, is robust to non-fasting conditions and triglyceride variability, making it practical for widespread clinical application.

Subgroup analyses confirmed a consistent NHHR–T2DM association across clinical strata, with only modest effect modification by marital status, smoking, alcohol use and chronic hepatitis B. These findings suggest that lifestyle factors and viral hepatitis status may amplify NHHR’s reflection of underlying metabolic dysregulation—consistent with prior work showing that alcohol intake modulates lipid-driven diabetes riskand that smoking impairs HDL functionality, attenuating its protective effects (40, 41). Although statistically significant interactions were observed for marital status and smoking, these factors are unlikely to represent biological effect modifiers per se. Rather, they may reflect differences in lifestyle patterns and HDL functionality that modestly modulate, but do not fundamentally alter, the association between NHHR and T2DM. Further, our mediation analyses—identifying fasting glucose and BMI as partial mediators of the NHHR–T2DM association—echo and extend findings from previous cohort studies. In the NAGALA cohort, complex lipid ratios including non-HDL-C/HDL-C mediated over 30% of the BMI-diabetes link, with RC/HDL-C showing the most substantial effect (~40%). Similarly, NAGALA data on BMI and NAFLD demonstrated that non-traditional lipid parameters (RC, RC/HDL-C, non-HDL-C/HDL-C, TC/HDL-C ratios) mediated upwards of 10% of the BMI-NAFLD association (22). Moreover, Mendelian randomization evidence suggests that part of the effect of BMI on CHD is mediated via elevated triglycerides and glycaemic dysregulation (42). Collectively, these data support our mechanistic inference that NHHR not only predicts cardiometabolic outcomes but also captures key lipid-driven pathways—namely, the interplay among insulin resistance, adiposity, and glucolipid metabolism—that underpin the transitions from NAFLD to T2DM (and potentially CHD).

This study has several limitations that should be considered when interpreting the magnitude of the observed associations. The retrospective observational design precludes causal inference, particularly whereby early dysglycemia may influence lipid profiles and thus overestimate the NHHR–T2DM association. Despite extensive adjustment, residual confounding from unmeasured factors such as diet, physical activity, medication use, and genetic susceptibility may persist, likely biasing effect estimates toward the null. NHHR was assessed only at baseline, and regression dilution from individual variability may further attenuate true associations. In addition, the lack of longitudinal assessment of NAFLD severity and the relatively limited number of CHD events, some of which were identified through administrative records, may have reduced statistical power and contributed to the weaker and heterogeneous CHD findings across cohorts. Although external validation in NHANES enhances robustness, the single-center origin of the primary cohort may limit generalizability. Future prospective, multi-center studies with repeated NHHR measurements, standardized outcome adjudication, and causal inference approaches are needed to refine effect estimation and clarify the clinical utility of NHHR in NAFLD. Clinically, NHHR offers a simple, cost-effective tool for risk stratification in NAFLD patients.

## Conclusion

This study demonstrates that NHHR is a readily available and economical lipid index for risk stratification of T2DM among patients with NAFLD. Elevated NHHR levels were robustly associated with incident T2DM across cohorts, supporting its clinical utility based on routine lipid panels. Although associations with CHD were less consistent, these findings underscore the complexity of cardiovascular risk prediction in NAFLD. Future prospective studies with repeated NHHR assessments and adjudicated outcomes are needed to clarify causality and define its role in integrated cardiometabolic risk management.

## Data Availability

The raw data supporting the conclusions of this article will be made available by the authors, without undue reservation.

## Footnotes

1
https://cks.nice.org.uk/topics/lipid-modification-cvd-prevention/

2 See https://www.cdc.gov/nchs/nhanes/index.htm.
