# Supplementary material for: Non-HDL-to-HDL cholesterol ratio predicts incident T2DM and CHD in non-alcoholic fatty liver disease: evidence from a large clinical cohort and NHANES
Source: Front Nutr. 2026 May 19;13:1750177. doi: 10.3389/fnut.2026.1750177 (PMC13226165; doi:10.3389/fnut.2026.1750177)
Supplement: Supplementary file 1 [file Supplementary_file_1.DOCX]

**Figure legends**

**Figure S1:** ROC curves of NHHR for predicting T2DM and CHD in the NHANES cohort

(A) ROC curves of NHHR for T2DM under three regression models. (B) ROC curves of NHHR for CHD under three regression models.


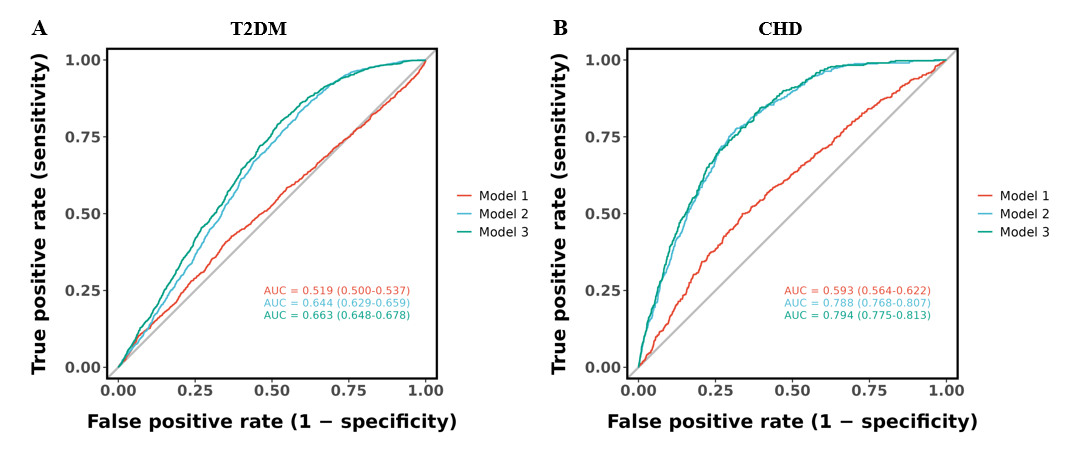


**Tables**

**Table S1 The baseline characteristics according to NHHR quartiles in the NHANES cohort**

| **Characteristic** | **Quartiles of NHHR** | | | | | **P-value** |
| --- | --- | --- | --- | --- | --- | --- |
|  | **Overall  N = 5,789** | **Q1˂ 2.5  N = 1,454** | **Q2(2.5-3.29)  N = 1,439** | **Q3(3.29- 4.31)  N = 1,455** | **Q4≥ 4.31  N = 1,441** |  |
| **Age (year)** | 53 ± 19 | 59 ± 19 | 54 ± 20 | 52 ± 18 | 49 ± 17 | <0.001 |
| **BMI (kg/m^2^)** | 33 ± 7 | 33 ± 7 | 34 ± 7 | 34 ± 7 | 33 ± 6 | <0.001 |
| **Sex** |  |  |  |  |  | <0.001 |
| Female | 2,221 (38.4%) | 721 (49.6%) | 646 (44.9%) | 492 (33.8%) | 362 (25.1%) |  |
| Male | 3,568 (61.6%) | 733 (50.4%) | 793 (55.1%) | 963 (66.2%) | 1,079 (74.9%) |  |
| **Marital Status** |  |  |  |  |  | <0.001 |
| Married | 3,792 (65.5%) | 869 (59.8%) | 940 (65.3%) | 989 (68.0%) | 994 (69.0%) |  |
| Single | 1,997 (34.5%) | 585 (40.2%) | 499 (34.7%) | 466 (32.0%) | 447 (31.0%) |  |
| **Ethnicity** |  |  |  |  |  | <0.001 |
| Hispanic | 2,191 (37.8%) | 476 (32.7%) | 538 (37.4%) | 581 (39.9%) | 596 (41.4%) |  |
| Non-Hispanic | 3,598 (62.2%) | 978 (67.3%) | 901 (62.6%) | 874 (60.1%) | 845 (58.6%) |  |
| **Hypertension** |  |  |  |  |  | <0.001 |
| No | 2,962 (51.2%) | 590 (40.6%) | 715 (49.7%) | 787 (54.1%) | 870 (60.4%) |  |
| Yes | 2,827 (48.8%) | 864 (59.4%) | 724 (50.3%) | 668 (45.9%) | 571 (39.6%) |  |
| **WBC (10^9^/L)** | 7.33 ± 2.04 | 7.21 ± 2.01 | 7.29 ± 1.93 | 7.26 ± 1.77 | 7.56 ± 2.38 | <0.001 |
| **RBC (10^12^/L)** | 4.85 ± 0.51 | 4.68 ± 0.51 | 4.80 ± 0.48 | 4.90 ± 0.51 | 5.01 ± 0.47 | <0.001 |
| **Hb (g/L)** | 14.56 ± 1.53 | 14.05 ± 1.48 | 14.38 ± 1.54 | 14.73 ± 1.50 | 15.08 ± 1.41 | <0.001 |
| **PLT (10^9^/L)** | 251 ± 69 | 244 ± 74 | 253 ± 68 | 254 ± 67 | 256 ± 68 | 0.001 |
| **ALT (U/L)** | 31 ± 34 | 27 ± 27 | 29 ± 21 | 32 ± 21 | 37 ± 55 | <0.001 |
| **AST (U/L)** | 27 ± 15 | 26 ± 18 | 26 ± 14 | 27 ± 11 | 29 ± 16 | <0.001 |
| **GGT (U/L)** | 41 ± 49 | 41 ± 62 | 38 ± 47 | 39 ± 40 | 45 ± 44 | <0.001 |
| **TC (mmol/L)** | 4.99 ± 1.11 | 4.20 ± 0.87 | 4.76 ± 0.87 | 5.14 ± 0.86 | 5.88 ± 1.11 | <0.001 |
| **TG (mmol/L)** | 1.98 ± 1.70 | 1.23 ± 0.52 | 1.62 ± 0.69 | 1.93 ± 0.82 | 3.17 ± 2.84 | <0.001 |
| **HDL-C (mmol/L)** | 1.17 ± 0.30 | 1.45 ± 0.33 | 1.22 ± 0.23 | 1.08 ± 0.18 | 0.93 ± 0.17 | <0.001 |
| **LDL-C (mmol/L)** | 2.98 ± 0.92 | 2.24 ± 0.64 | 2.82 ± 0.70 | 3.20 ± 0.75 | 3.68 ± 0.89 | <0.001 |
| **Glu (mmol/L)** | 6.59 ± 2.60 | 6.67 ± 2.43 | 6.35 ± 2.12 | 6.50 ± 2.49 | 6.83 ± 3.20 | <0.001 |
| **CHD** |  |  |  |  |  | <0.001 |
| No | 5,379 (92.9%) | 1,300 (89.4%) | 1,340 (93.1%) | 1,364 (93.7%) | 1,375 (95.4%) |  |
| Yes | 410 (7.1%) | 154 (10.6%) | 99 (6.9%) | 91 (6.3%) | 66 (4.6%) |  |
| **T2DM** |  |  |  |  |  | 0.017 |
| No | 4,495 (77.6%) | 1,089 (74.9%) | 1,132 (78.7%) | 1,157 (79.5%) | 1,117 (77.5%) |  |
| Yes | 1,294 (22.4%) | 365 (25.1%) | 307 (21.3%) | 298 (20.5%) | 324 (22.5%) |  |

All the variables are presented as the mean (SE) or n (%). Abbreviations: NHHR, non-high-density lipoprotein cholesterol to high-density lipoprotein cholesterol ratio; BMI, body mass index; WBC​​, White Blood Cell Count; ​​RBC​​, Red Blood Cell Count; Hb​​, Hemoglobin; ​​PLT,​​ Platelet Count; ​​ALT​​, Alanine Aminotransferase​​; AST​​, Aspartate Aminotransferase; GGT​​, γ-Glutamyl Transferase​​; TC​​, Total Cholesterol​​; TG​​, Triglyceride​​; HDL-C, High-Density Lipoprotein Cholesterol; LDL-C, Low-Density Lipoprotein Cholesterol; Glu​​, Glucose; ​CHD,​​ Coronary Heart Disease; T2DM,​​Type 2 Diabetes Mellitus.

**Table S2** **The correlation between NHHR and T2DM/CHD in NAFLD populations in the NHANES cohort**

| **Characteristic** | **Model 1** | | | **Model 2** | | | **Model 3** | | |  |
| --- | --- | --- | --- | --- | --- | --- | --- | --- | --- | --- |
|  | **OR** | **95% CI** | **P-value** | **OR** | **95% CI** | **P-value** | **OR** | **95% CI** | **P-value** |  |
| **T2DM** |  |  |  |  |  |  |  |  |  |  |
| **NHHR (continuous)** | 1.00 | 0.96, 1.04 | 0.980 | 1.09 | 1.04, 1.14 | <0.001 | 1.09 | 1.04, 1.14 | <0.001 |  |
| **NHHR (quartile)** |  |  |  |  |  |  |  |  |  |  |
| Q1 | 1(Reference) | 1(Reference) |  | 1(Reference) | 1(Reference) |  | 1(Reference) | 1(Reference) |  |  |
| Q2 | 0.80 | 0.67, 0.95 | 0.010 | 0.93 | 0.78, 1.12 | 0.452 | 0.92 | 0.77, 1.10 | 0.351 |  |
| Q3 | 0.76 | 0.64, 0.91 | 0.002 | 0.97 | 0.81, 1.17 | 0.778 | 0.96 | 0.80, 1.15 | 0.663 |  |
| Q4 | 0.86 | 0.72, 1.02 | 0.076 | 1.21 | 1.01, 1.46 | 0.038 | 1.21 | 1.01, 1.46 | 0.043 |  |
| P for trend |  |  | 0.063 |  |  | 0.043 |  |  | 0.049 |  |
| **CHD** |  |  |  |  |  |  |  |  |  |  |
| **NHHR (continuous)** | | 0.79 | 0.73, 0.86 | <0.001 | 0.90 | 0.83, 0.98 | 0.012 | 0.90 | 0.83, 0.98 | 0.021 |
| **NHHR (quartile)** | |  |  |  |  |  |  |  |  |  |
| Q1 | | 1(Reference) | 1(Reference) |  | 1(Reference) | 1(Reference) |  | 1(Reference) | 1(Reference) |  |
| Q2 | | 0.62 | 0.47, 0.80 | <0.001 | 0.77 | 0.58, 1.01 | 0.062 | 0.76 | 0.58, 1.01 | 0.055 |
| Q3 | | 0.56 | 0.43, 0.74 | <0.001 | 0.76 | 0.57, 1.01 | 0.057 | 0.76 | 0.57, 1.02 | 0.068 |
| Q4 | | 0.40 | 0.30, 0.54 | <0.001 | 0.62 | 0.45, 0.85 | 0.003 | 0.64 | 0.46, 0.88 | 0.006 |
| P for trend | |  |  | <0.001 |  |  | 0.003 |  |  | 0.006 |

Abbreviations: NHHR, non-high-density lipoprotein cholesterol to high-density lipoprotein cholesterol ratio; T2DM,​​Type 2 Diabetes Mellitus; CHD,​​ Coronary Heart Disease; CI, Confidence Interval; OR, Odds Ratio.

Model 1: no covariates were adjusted.

Model 2: adjusted for sex, and age.

Model 3: adjusted for sex, age, BMI, marital status.
